# Clinical Study of Anlotinib as Third-Line or Above Therapy in Patients With Advanced or Metastatic Gastric Cancer: A Multicenter Retrospective Study

**DOI:** 10.3389/fonc.2022.885350

**Published:** 2022-07-04

**Authors:** Caiyun Nie, Yunduan He, Huifang Lv, Ming Gao, Xiaohui Gao, Beibei Chen, Weifeng Xu, Jianzheng Wang, Yingjun Liu, Jing Zhao, Xiaobing Chen

**Affiliations:** ^1^ Department of Medical Oncology, Affiliated Cancer Hospital of Zhengzhou University, Henan Cancer Hospital, Zhengzhou, China; ^2^ State Key Laboratory of Esophageal Cancer Prevention & Treatment, Zhengzhou University, Zhengzhou, China; ^3^ Henan Engineering Research Center of Precision Therapy of Gastrointestinal Cancer, Zhengzhou, China; ^4^ Zhengzhou Key Laboratory for Precision Therapy of Gastrointestinal Cancer, Zhengzhou, China; ^5^ Department of Oncology, First Affiliated Hospital of Zhengzhou University, Zhengzhou, China; ^6^ Department of Oncology, First Affiliated Hospital of Henan University of Science and Technology, Luoyang, China; ^7^ Department of General Surgery, Affiliated Cancer Hospital of Zhengzhou University, Henan Cancer Hospital, Zhengzhou, China

**Keywords:** gastric cancer, anlotinib, anti-angiogenesis, targeted therapy, efficacy

## Abstract

**Background:**

The present study was conducted to evaluate the efficacy and safety of anlotinib as third-line or above therapy for patients with advanced or metastatic gastric cancer.

**Methods:**

Patients with advanced or metastatic gastric cancer who have failed from second-line treatment and treated with anlotinib monotherapy or combined with chemotherapy or immunotherapy from June 2019 to January 2021 in 3 institutions across China were retrospectively analyzed. The primary end point was progression free survival (PFS). Secondary end points included overall survival (OS), objective response rate (ORR), disease control rate (DCR), and safety.

**Results:**

43 patients with advanced or metastatic gastric cancer who have failed prior treatment received anlotinib monotherapy or combination therapy as third-line or above therapy. In the general population, 4 patients achieved PR, 21 patients had SD and 18 patients had PD. The overall ORR and DCR were 9.3% (4/43) and 58.1% (25/43), respectively. Median PFS and OS were 3.0 months (95% CI=2.5-3.5) and 6.0 months (95% CI=4.4-7.6), respectively. The incidence of Grade 3-4 adverse events(AEs) was 34.9%. Subgroup analysis suggested that the ORR of anlotinib combination therapy was superior than anlotinib monotherapy, but with similar PFS and OS. The clinical benefit of anlotinib was not associated with previously anti-angiogenesis therapy with apatinib.

**Conclusions:**

Anlotinib monotherapy or combination therapy provide a feasible third-line or above therapeutic strategy in patients with advanced or metastatic gastric cancer a median PFS of 3.0 months and median OS of 6.0 months was obtained with well tolerated toxicity.

## Introduction

According to the latest global cancer burden data in 2020, gastric cancer is currently the fifth most common cancer type and the fourth leading cause of death in the world (1). In 2020, there were 1.089 million new cases of gastric cancer and 768,000 deaths worldwide, of which 478,000 were new cases of gastric cancer in China, and 373,000 (48.5%) died of gastric cancer. China is the country with the largest number of gastric cancer incidence and deaths in the world. Most gastric cancer patients are in advanced stage when diagnosed, and the prognosis is still poor.

Gastric cancer has entered the era of targeted therapy and immunotherapy with the continuous deepening of understanding of its biological behavior (2–4). The most effective targeted therapy of gastric cancer is still focused on the anti-human epidermal growth factor receptor-2 (HER2) and anti-vascular endothelial growth factor (VEGF) pathway. As an oral highly selective small molecule tyrosinase inhibitor of VEGFR2, apatinib has been approved for third-line treatment indications for advanced gastric cancer in China (5).

Anlotinib is a novel oral small molecule multi-targeted tyrosine kinase inhibitor (TKI) with proven efficacy in many solid tumors, which can effectively inhibit vascular endothelial growth factor receptor (VEGFR), platelet-derived growth factor (PDGFR), fibroblast growth factor receptor (FGFR), c-Kit and other kinases (6, 7). Anlotinib has been approved by the China Food and Drug Administration (CFDA) for advanced non-small cell lung cancer (NSCLC) and soft tissue sarcoma (8–10). Meanwhile, real-world studies have also shown that anlotinib is effective in a variety of solid tumors, including gastric cancer (11). This multicenter retrospective and observational study aimed to evaluate the efficacy and safety of anlotinib for patients with advanced or metastatic gastric cancer as third-line or above therapy.

## Methods

### Patients Population

From June 2019 to January 2021, data of patients with advanced or metastatic gastric cancer who have failed prior treatment and treated with anlotinib monotherapy or combination therapy as third-line or above therapy in 3 institutions across China was retrospectively collected in this study, including the Affiliated Cancer Hospital of Zhengzhou University, First Affiliated Hospital of Zhengzhou University and First Affiliated Hospital of Henan University of Science and Technology.

### Study Treatment

In this study, the patients received anlotinib monotherapy or combination therapy until disease progression, unacceptable toxicity or death. In the monotherapy regimen, anlotinib was given at a dose of 10 mg or 12mg once a day on d1 to d14 every three weeks. In the combination therapy regimen, anlotinib was given at a dose of 10 mg once a day on d1 to d14 every three weeks, and concurrent chemotherapy or immunotherapy was given simultaneously. Among them, the chemotherapy regimen includes docetaxel and albumin bound paclitaxel, and the immunotherapy regimen includes sintilimab and camrelizumab.

### Efficacy and Safety Assessments

After treatment, all patients underwent imaging examination every two cycles to evaluate the clinical efficacy. The efficacy evaluation criteria are RECIST version 1.1 response evaluation criteria in solid tumors, including complete response (CR), partial response (PR), stable disease (SD), and progressive disease (PD). The objective response rate (ORR) was CR + PR, and the disease control rate (DCR) was CR+ PR and SD. Adverse events (AEs) were assessed according to the Common Terminology Criteria for Adverse Events, version 4.0.

### Statistical Analysis

Survival curves of patients were estimated by the Kaplan-Meier method. The follow-up deadline is June 1, 2021. Progression-free survival (PFS) was defined as starting anlotinib monotherapy or combination therapy to disease progression or death. Overall survival (OS) was defined as the period from the time of treatment with anlotinib monotherapy or combination therapy to patient death or last follow-up. All the statistical descriptive analyses were performed with SPSS 22.0 software (SPSS Inc., IL, US) software. P<0.05 was considered significant.

## Results

### Patient and Treatment Characteristics

A total of 43 patients with advanced or metastatic gastric cancer were included in the present study. Patient and treatment characteristics are summarized in **Table 1**. The median age was 59 years (range 35-74), with 8 female patients and 35 male patients. Thirty-seven patients had advanced gastric cancer, and the other 6 patients had gastroesophageal junction (GEJ) adenocarcinoma. All the patients were diagnosed as advanced or recurrent, common metastatic sites included lymph node (62.8%), peritoneum (41.9%), liver (37.2%), and lung (23.3%). All the patients in the study had undergone prior systemic therapies, anlotinib was given as third line treatment in 24 patients (55.8%) and fourth to eighth-line treatment in 19 patients (44.2%). 28 patients had previously received anti-angiogenesis therapy with apatinib. Five patients received anlotinib monotherapy, and the other 38 cases received anlotinib combination therapy. Of these cases, 14 patients received anlotinib combined with chemotherapy, 20 cases received anlotinib combined with PD-1 inhibitor, and the other 4 patients received a combination of anlotinib, chemotherapy and PD-1 inhibitor.

**Table 1:** Patient and treatment characteristics.

Characteristic	Total (n=43) n (%)	Monotherapy (n=5) n (%)	Combination therapy(n=38)n (%)
**Age (years, median)**	59	57	59
**Gender**			
Female	8 (18.6)	0 (0)	8 (21.1)
Male	35 (81.4)	5 (100)	30 (78.9)
**ECOG**			
0-1	30 (69.8)	3 (60.0)	27 (71.1)
2	13 (30.2)	2 (40.0)	11 (28.9)
**Primary tumor site**			
Gastric	37 (86.0)	3 (60.0)	34 (89.5)
GEJ	6 (14.0)	2 (40.0)	4 (10.5)
**Metastatic site**			
Lymph node	27 (62.8)	3 (60.0)	24 (63.2)
Peritoneum	18 (41.9)	0 (0)	18 (47.4)
Liver	16 (37.2)	3 (60.0)	13 (34.2)
Lung	10 (23.3)	1 (20.0)	9 (23.7)
Others	14 (32.6)	1 (20.0)	13 (34.2)
**Number of metastatic sites**			
**1-2**	31 (72.1)	5 (100)	26 (68.4)
**≥ 3** **Treatment line**	12 (27.9)	0 (0)	12 (31.6)
**3**	24 (55.8)	2 (40.0)	22 (57.9)
**≥ 4**	19 (44.2)	3 (60.0)	16 (42.1)
**Prior apatinib therapy**			
**Yes**	28 (65.1)	5 (100)	23 (60.5)
**No**	15 (34.9)	0 (0)	15 (39.5)

ECOG, Eastern Cooperative Oncology Group performance status; GEJ, Gastroesophageal Junction Tumors.

### Efficacy

**Figure 1:**
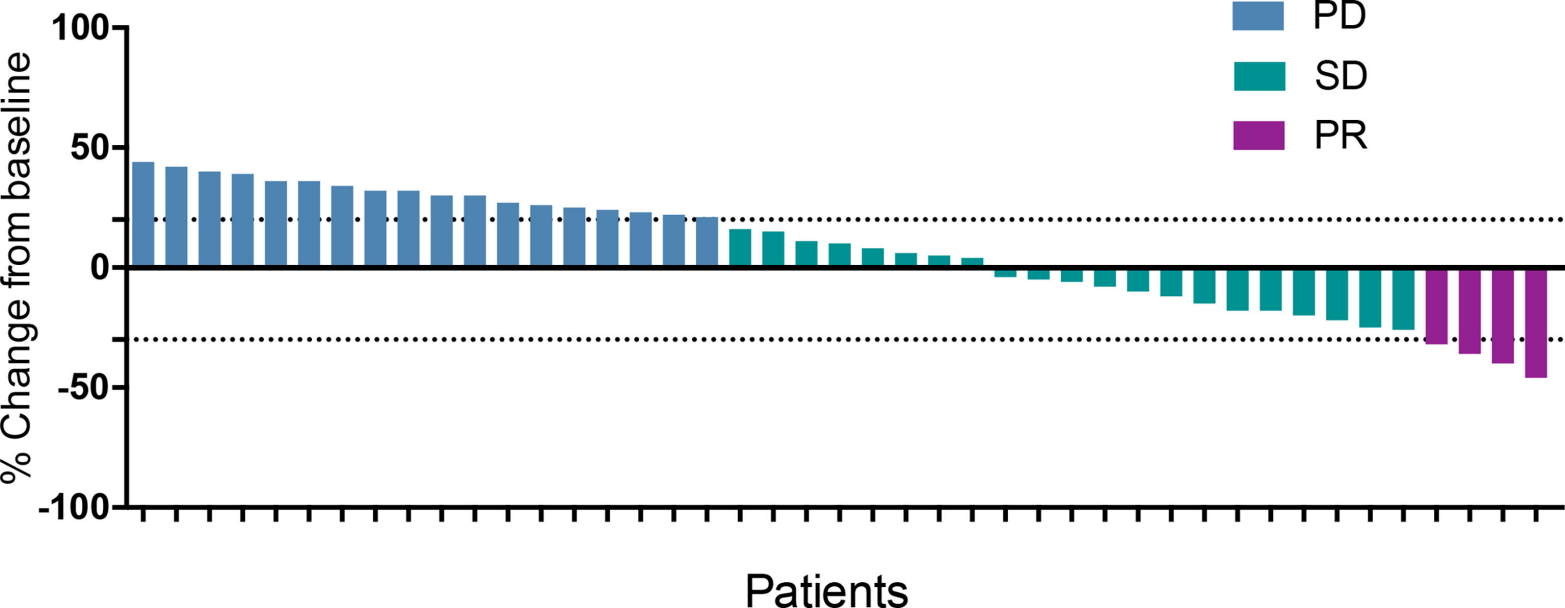
Waterfall plot of the best response change.

In the general population, CR was not observed, 4 patients achieved PR, 21 patients had SD and 18 patients had PD. The waterfall plot of the best response change was shown in **Figure 1**. The overall ORR and DCR were 9.3% (4/43) and 58.1% (25/43), respectively (**Table 2**). In the anlotinib monotherapy population, 3 patients had SD and 2 patients had PD. The overall ORR and DCR were 0% (0/5) and 60.0% (3/5), respectively. In the combination therapy population, 4 patients achieved PR, 18 patients had SD and 16 patients had PD. The overall ORR and DCR were 10.5% (4/38) and 57.9% (22/38), respectively. In the anlotinib combined with chemotherapy, 1 patient achieved PR, 10 patients had SD and 3 patients had PD. The overall ORR and DCR were 7.1% (1/14) and 78.6% (11/14), respectively. In the anlotinib combined with PD-1 inhibitor, 3 patient achieved PR, 7 patients had SD and 10 patients had PD. The overall ORR and DCR were 15.0% (3/20) and 50.0% (10/20), respectively. In patients who had previously received anti-angiogenesis therapy, 3 patients achieved PR, 14 patients had SD and 11 patients had PD. The overall ORR and DCR were 10.7% (3/28) and 60.7% (17/28), respectively. In patients who had not previously received anti-angiogenesis therapy, 1 patient achieved PR, 7 patients had SD and 7 patients had PD. The overall ORR and DCR were 6.7% (1/15) and 53.3% (8/15), respectively.

**Table 2:** Efficacy of anlotinib in patients with advanced or metastatic gastric cancer.

Parameter	Best response				ORR	DCR	Median PFS (95%CI)	Median OS (95%CI)
	CR	PR	SD	PD				
Total	0	4	21	18	9.3% (4/43)	58.1% (25/43)	3.0(2.5-3.5)	6.0(4.4-7.6)
Treatment programs								
Monotherapy	0	0	3	2	0% (0/5)	60.0% (3/5)	3.5(0-8.1)	6.2(3.6-8.8)
Combination	0	4	18	16	10.5% (4/38)	57.9% (22/38)	3.0(2.5-3.5)	6.0(4.2-7.8)
Combination type								
Chemotherapy	0	1	10	3	7.1% (1/14)	78.6% (11/14)	3.2(2.8-3.6)	9.0(4.4-13.6)
PD-1 inhibitor	0	3	7	10	15.0% (3/20)	50.0% (10/20)	3.0(1.9-4.1)	5.0(3.9-6.1)
Prior apatinib therapy								
Yes	0	3	14	11	10.7% (3/28)	60.7% (17/28)	3.0(2.1-3.9)	6.0(2.8-9.2)
No	0	1	7	7	6.7% (1/15)	53.3% (8/15)	3.0(1.9-4.1)	5.0(2.6-7.4)

CR, complete response; PR, partial response; SD, stable disease; PD, progressive disease; ORR, overall response rate; DCR, disease control rate; PFS, progression free survival; OS, overall survival.

**Figure 2:**
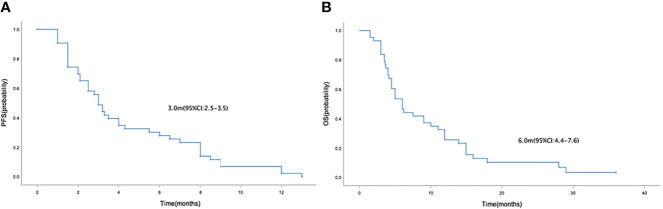
Kaplan-Meier curve of PFS **(A)** and OS **(B)** in the general population.

**Figure 3:**
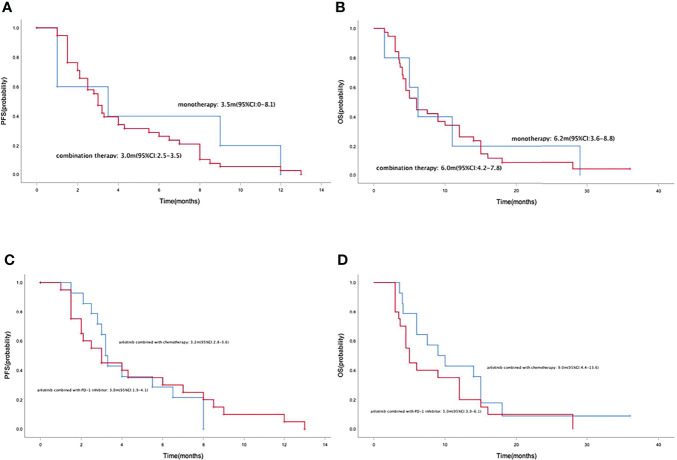
Kaplan-Meier curve of PFS **(A)** and OS **(B)** in anlotinib monotherapy and combination therapy population. Kaplan-Meier curve of PFS **(C)** and OS **(D)** in anlotinib combined with chemotherapy and PD-1 inhibitor population.

**Figure 4:**
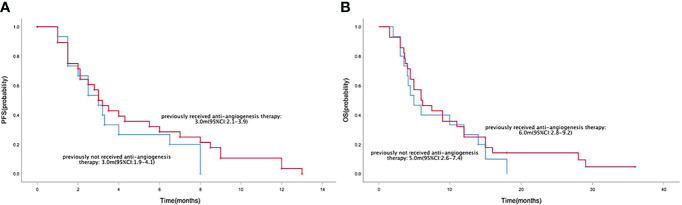
Kaplan-Meier curve of PFS **(A)** and OS **(B)** in the patients who had or not previously received anti-angiogenesis therapy population.

Median PFS and OS in the 43 patients with advanced or metastatic gastric cancers were 3.0 months (95% CI=2.5-3.5) (**Figure 2A**) and 6.0 months (95% CI=4.4-7.6) (**Figure 2B**), respectively. Median PFS in the anlotinib monotherapy and combination therapy population were 3.5 months (95% CI=0-8.1) and 3.0 months (95% CI=2.5-3.5) (**Figure 3A**), respectively. Median OS in the anlotinib monotherapy and combination therapy population were 6.2 months (95% CI=3.6-8.8) and 6.0 months (95% CI=4.2-7.8) (**Figure 3B**) respectively. Median PFS in the anlotinib combined with chemotherapy and PD-1 inhibitor population were 3.2 months (95% CI=2.8-3.6) and 3.0 months (95% CI=1.9-4.1) (**Figure 3C**) respectively. Median OS was 9.0 months (95% CI=4.4-13.6) and 5.0 months (95% CI=3.9-6.1) (**Figure 3D**), respectively. Median PFS in the patients who had or not previously received anti-angiogenesis therapy were 3.0 months (95% CI=2.1-3.9) and 3.0 months (95% CI=1.9-4.1) (**Figure 4A**), respectively. Median OS was 6.0 months (95% CI=2.8-9.2) and 5.0 months (95% CI=2.6-7.4) (**Figure 4B**), respectively.

### Safety

All the patients developed at least one treatment-related adverse event (TRAE) and most of the adverse events were grade 1-2 in severity(**Table 3**). The incidence of Grade 3-4 AEs was 34.9%. No unexpected side effects or treatment-related death were observed. The most common anlotinib monotherapy or combination treatment-related non-hematological AEs were fatigue (n=19, 44.2%), secondary hypertension (n=16, 37.2%), hand-foot syndrome (n=14, 32.6%), anorexia (n=14, 32.6%), muscle pain/joint pain (n=13, 30.2%), proteinuria (n=11, 25.6%), diarrhea(n=11, 25.6%), nausea or vomiting (n=9, 20.9%), rash (n=8, 18.6%), oral mucositis (n=6, 14.0%), hypothyroidism (n=5, 11.6%), sensory neurotoxicity (n=3, 7.0%) and pneumonitis (n=3, 7.0%). Hematological anlotinib monotherapy or combination treatment-related AEs were decreased neutrophil count (n=15, 34.9%), decreased white blood count (n=15, 34.9%), anemia (n=11, 25.6%), decreased platelet (n=5, 11.6%), increased ALT/AST (n=10, 23.3%) and hyperbilirubinemia (n=4, 9.3%). Grade 3-4 AEs were secondary hypertension, proteinuria, muscle pain/joint pain, decreased neutrophil and white blood count and increased ALT/AST.

**Table 3:** Treatment-related adverse events (TRAEs).

Adverse Event	All Grade n(%)	≥ Grade3 n(%)
Non- Hematologic		
Secondary hypertension	16 (37.2)	2 (4.7)
Hand-foot syndrome	14 (32.6)	0
Fatigue	19 (44.2)	0
Rash	8 (18.6)	0
Pneumonitis	3 (7.0)	0
Sensory neurotoxicity	3 (7.0)	0
Proteinuria	11 (25.6)	2 (4.7)
Oral mucositis	6 (14.0)	0
Nausea or Vomiting	9 (20.9)	0
Diarrhea	11 (25.6)	0
Hypothyroidism	5 (11.6)	0
Muscle pain/joint pain	13 (30.2)	1 (2.3)
Anorexia	14 (32.6)	0
Hematologic		
Decreased neutrophil count	15 (34.9)	511.6)
Decreased white blood count	15 (34.9)	5 (11.6)
Anemia	11 (25.6)	0
Decreased platelet	5 (11.6)	0
Increased ALT/AST	10 (23.3)	2 (4.7)
Hyperbilirubinemia	4 (9.3)	0

## Discussion

Gastric cancer has become a malignant tumor that seriously threatens human health. The treatment methods and drugs of gastric cancer have experienced many changes. Before the 1960s, the treatment of advanced gastric cancer was mainly supportive treatment. Since then, it was found that patients with advanced gastric cancer can benefit from chemotherapy (12, 13). Over the past few decades, chemotherapy drugs and regimens were constantly updated and improved, and chemotherapy has been the cornerstone treatment of advanced gastric cancer and has played an important role until now. However, the current efficacy of traditional chemotherapy drugs has reached a plateau stage, which is difficult to further improve the prognosis of advanced or metastatic gastric cancer patients. A number of small-sample phase II studies and retrospective studies have explored the efficacy of chemotherapy in third-line patients with advanced gastric cancer, including docetaxel, irinotecan and FOLFIRI regimens, which have very limited survival benefit in the third-line treatment (14, 15). Therefore, in order to improve the treatment status of patients with advanced or metastatic gastric cancer, targeted therapy and immunotherapy have become new strategies.

HER2 is the first and most important target of gastric cancer, and the ToGA study confirmed that trastuzumab combined with chemotherapy can significantly prolong the overall survival of patients with HER2-positive advanced or metastatic gastric cancer (2). The expression rate of HER2 in gastric cancer is about 15%, and most patients are HER2 negative (16, 17). However, for the HER2-negative gastric cancer population, despite continuous exploration in the following 10 years, most clinical studies of targeted drugs ended in failure. In 2014, the results of the global multicenter phase III randomized clinical trial RAINBOW showed that ramucirumab combined with paclitaxel significantly improved the PFS and was approved by the FDA for the second-line treatment of gastric cancer (18). Subsequently, the RAINBOW-Asia study confirmed the efficacy and safety of this regimen in Asian populations (19). However, ramucirumab has not yet been marketed in China. As a tyrosine kinase inhibitor that selectively inhibits the vascular endothelial growth factor receptor- 2 (VEGFR2), apatinib has been approved in patients with advanced or metastatic adenocarcinoma of the stomach or GEJ as third- or further- line treatment in China (5).

However, the efficacy of other anti-angiogenic drugs still needs to be explored. Anlotinib is a novel oral small molecule multi-targeted TKI with proven efficacy in many solid tumors (20, 21). In the present study, 43 patients with advanced or metastatic gastric cancer, the overall ORR and DCR were 9.3% (4/43) and 58.1% (25/43), and a median PFS of 3.0 months and median OS of 6.0 months was obtained. Phase III clinical study demonstrated that apatinib treatment significantly improved median PFS (2.6 months vs. 1.8 months, p=0.016) and disease control rate (42.05% vs. 8.79%, p<0.016) compared with the placebo group (5). But it is worth noting that all the patients received apatinib as third-line treatment in the above phase III study. In contrast, the proportion of patients who received anlotinib as third-line therapy in our present study was only 55.8%, the other 44.2% patients received anlotinib as fourth to eighth-line treatment. In such a patient population that included more late-line treatments, anlotinib still achieved very good clinical efficacy, which shows the clinical value of anlotinib in the third-line and above therapy of advanced or metastatic gastric cancer.

The optimal drug treatment model of anti-angiogenesis therapy for gastric cancer is still inconclusive. In this study, 5 patients received anlotinib monotherapy, and the other 38 patients received anlotinib combination therapy. The ORR of the combined treatment group were higher than those of the monotherapy group. Combination with chemotherapy or in combination with immunotherapy are effective ways of antiangiogenic therapy (22). On the one hand, anti-angiogenic therapy can modulate the immune microenvironment, and can play a synergistic effect in combination with immunotherapy (23, 24); on the other hand, anti-angiogenic therapy can restore the normalization of local blood vessels, enabling chemotherapeutic drugs to reach the tumor site to exert their role (25). However, there were no statistically significant differences in PFS and OS in the combination therapy group compared with the monotherapy group, therefore the optimal mode of anti-angiogenic therapy for gastric cancer still needs to be explored. As only 5 patients were treated with anlotinib monotherapy, in order to better explain the therapeutic effect of anlotinib monotherapy, we also compared the efficacy of anlotinib monotherapy with that of chemotherapy. In the same period, 20 patients received irinotecan single drug chemotherapy. The ORR and DCR were 10% and 35%, and the median PFS and OS were 2.1 months (1.8-2.4) and 5.6 months (4.9-6.3), respectively. Anlotinib monotherapy had higher DCR and better PFS, OS. With respect to drug safety, anlotinib monotherapy had a lower incidence of AEs. For patients with poor ECOG scores, anlotinib monotherapy could be an optional strategy in terms of safety profile.

Anti-angiogenic therapy has now become an important treatment strategy for advanced gastric cancer. In the third-line and later-line therapy, most patients may previously have received anti-angiogenic drug therapy (26). In this study, 65.1% patients had previously received apatinib therapy, the efficacy was similar between prior and apatinib-naïve groups. Previous apatinib therapy did not affect the clinical benefit of anlotinib. Anlotinib provides a new treatment option for patients who have failed from prior antiangiogenic therapy with apatinib.

Most of the adverse events observed in this study were grade 1-2 in severity, which could be relieved by symptomatic treatment. Grade 3-4 adverse reactions occurred in 15 (34.9%) patients. Anlotinib related AEs were consistent with the known safety profiles of anti- angiogenesis therapy, including secondary hypertension, hand-foot syndrome and proteinuria (27–29). Most AEs mainly come from chemotherapy and immunotherapy.

There are some limitations in our study, because it is a retrospective study, and the number of cases is not sufficiently large. Thus, large randomized controlled trials are needed to confirm the clinical value of anlotinib for patients with advanced or metastatic gastric cancer as third-line or above therapy.

## Conclusion

Anlotinib monotherapy or combination therapy provide a feasible third-line or above therapeutic strategy in patients with advanced or metastatic gastric cancer a median PFS of 3.0 months and median OS of 6.0 months was obtained with well tolerated toxicity.

## Data Availability Statement

The original contributions presented in the study are included in the article/supplementary material. Further inquiries can be directed to the corresponding author.

## Ethics Statement

The studies involving human participants were reviewed and approved by The ethics committee of the Affiliated Cancer Hospital of Zhengzhou University(2021-KY-0192). The patients/participants provided their written informed consent to participate in this study.

## Author Contributions

CN and XC designed the research, analyzed the data and drafted the paper. CN, YH, HL, MG, XG, BC and WX were mainly responsible for data collection and analysis. CN, JW, YL and JZ were primarily responsible for statistical analysis. All authors contributed to the article and approved the submitted version.

## Funding

This work was supported by Medical Science and Technique Foundation of Henan Province (No. 212102310623), 1000 Talents Program of Central plains (No. 204200510023), Medical Science and Technique Foundation of Henan Province (No. SB201901101), Young and Middle-aged Health and Technology Innovation Leading Talent Project of Henan Province (No. YXKC2020008) and the Sate Key Laboratory of Esophageal Cancer Prevention & Treatment (No. Z2020000X).

## Conflict of Interest

The authors declare that the research was conducted in the absence of any commercial or financial relationships that could be construed as a potential conflict of interest.

## Publisher’s Note

All claims expressed in this article are solely those of the authors and do not necessarily represent those of their affiliated organizations, or those of the publisher, the editors and the reviewers. Any product that may be evaluated in this article, or claim that may be made by its manufacturer, is not guaranteed or endorsed by the publisher.
